# Supplementary material for: Boosting GWAS using biological networks: A study on susceptibility to familial breast cancer
Source: PLoS Comput Biol. 2021 Mar 18;17(3):e1008819. doi: 10.1371/journal.pcbi.1008819 (PMC8009366; doi:10.1371/journal.pcbi.1008819)
Supplement: S3 Table — The first row within each block contains the summary statistics on the whole network. (PDF) [file pcbi.1008819.s003.pdf]

Network	SNPs	Edges	Subnetworks	$\overline{\text{Betweenness}}$	$\hat{\text{P}}_{\text{SNP}}$
GS	197 083	197 060	-	1.04×10^{-3}	0.49
SConES GS	1 590	1 585	5	1.30×10^{-3}	0.023
GM	197 083	6 442 446	-	2.05×10^{-4}	0.49
SConES GM	1 692	177 611	5	2.27×10^{-4}	0.055
GI	197 083	28 733 720	-	7.50×10^{-5}	0.49
SConES GI	408	539	5	4.81×10^{-4}	0.076

$\overline{\text{Betweenness}}$: mean (normalized) betweenness of the selected SNPs in the corresponding full network.$\hat{\text{P}}_{\text{SNP}}$: median P-value of the selected SNPs.
