# Supplementary figures and images for: Boosting GWAS using biological networks: A study on susceptibility to familial breast cancer
Source: PLoS Comput Biol. 2021 Mar 18;17(3):e1008819. doi: 10.1371/journal.pcbi.1008819 (PMC8009366; doi:10.1371/journal.pcbi.1008819)

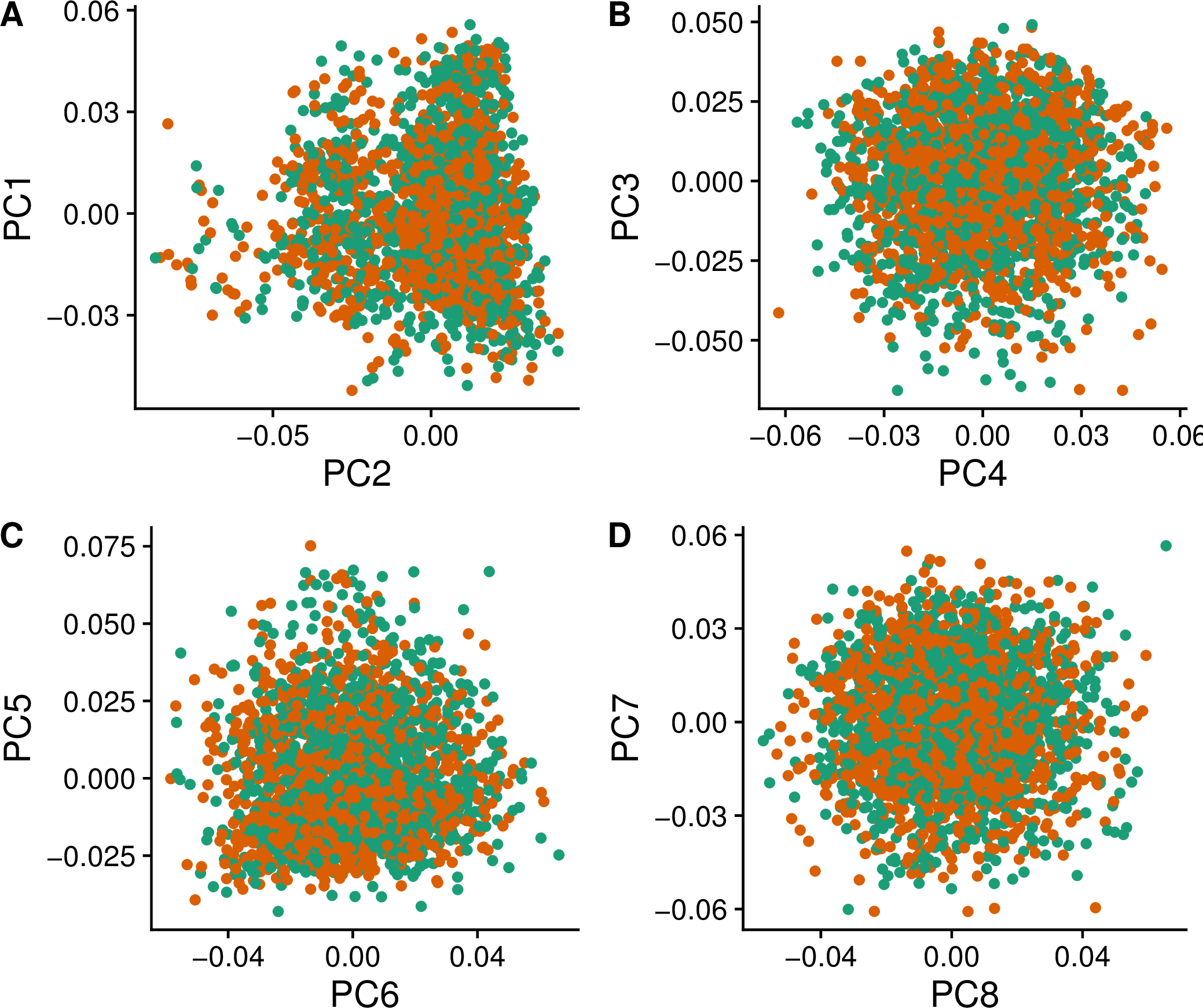

Supplement: S1 Fig — (A,B,C,D) Eight main principal components, computed on the genotypes of GENESIS. Cases are colored in green, controls in orange. (TIF) [file pcbi.1008819.s008.tif]

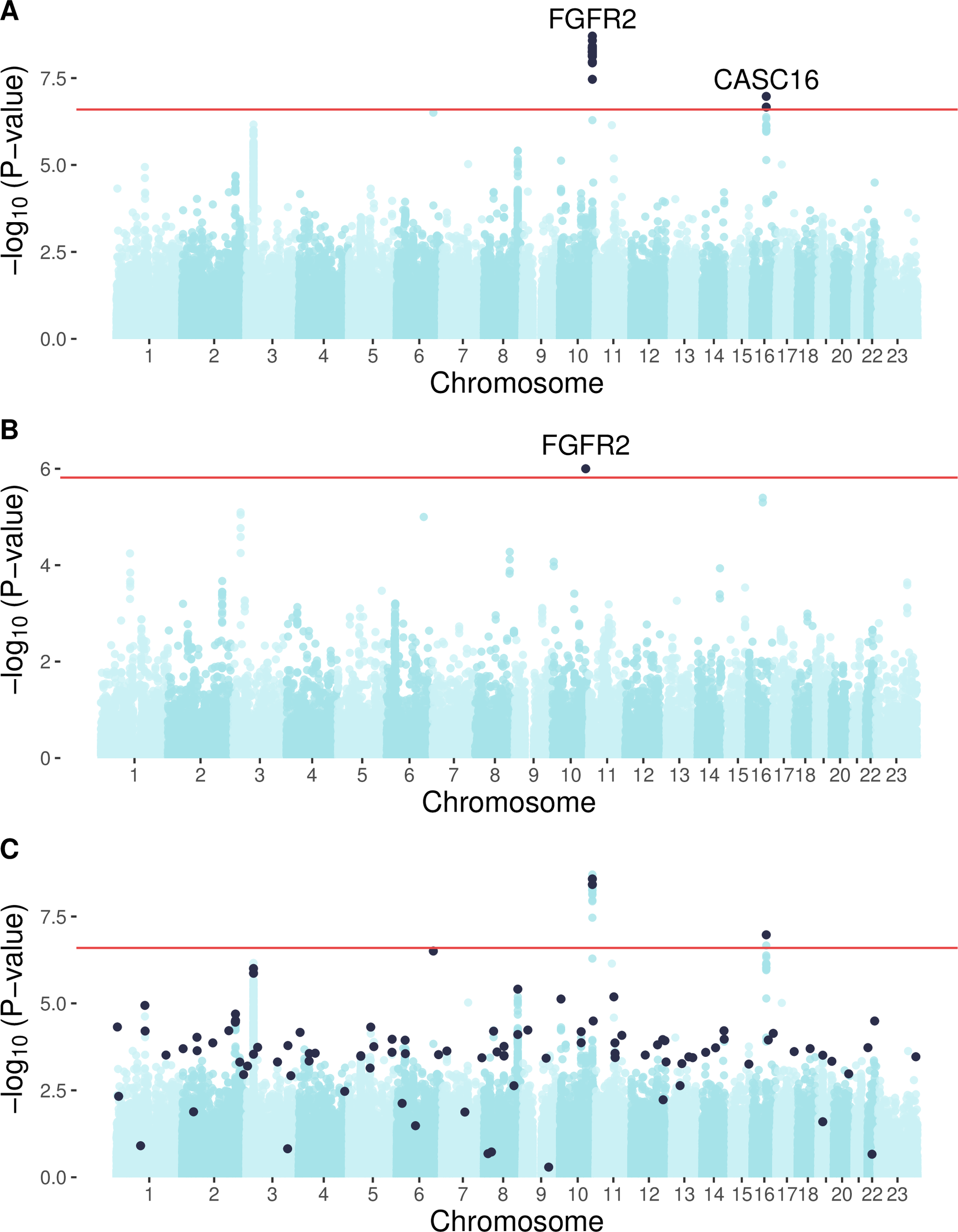

Supplement: S2 Fig — (A) SNP association, measured from the outcome of a 1 d.f. χ2 allelic test (Section 2.2). Significant SNPs within a coding gene, or within 50 kilobases of its boundaries, are annotated. The Bonferroni threshold is 2.54 × 10^-7^. (B) Gene association, measured by P-value of VEGAS2 [22] using the 10% of SNPs with the lowest P-values (Section 2.2). The Bonferroni threshold is 1.53 × 10^-6^. (C) SNP association as in panel (A). The SNPs in black were selected by an L1-penalized logistic regression (Section 2.5.2, λ = 0.03). (TIF) [file pcbi.1008819.s009.tif]

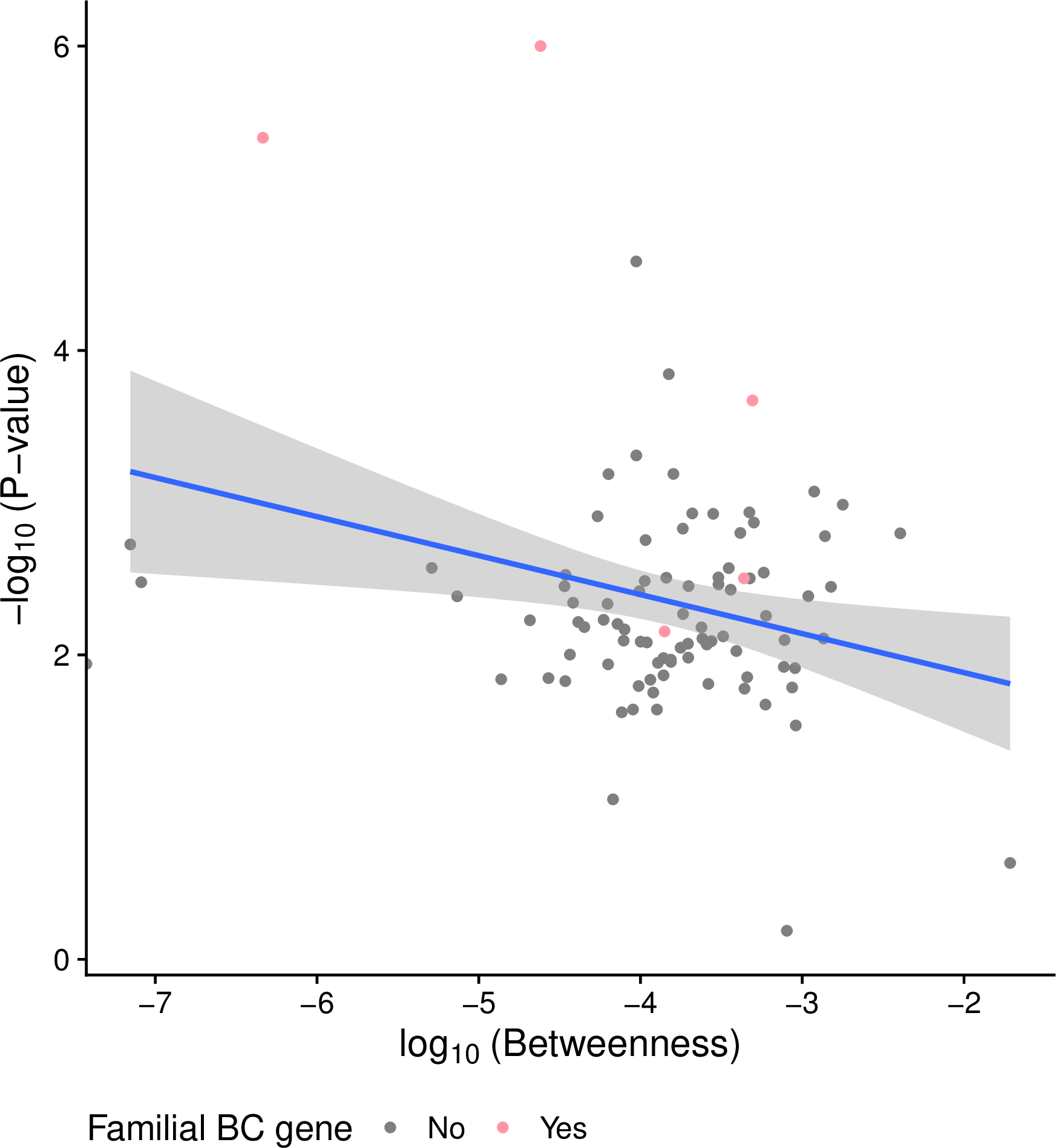

Supplement: S3 Fig — The blue line represents a fitted generalized linear model. (TIF) [file pcbi.1008819.s010.tif]

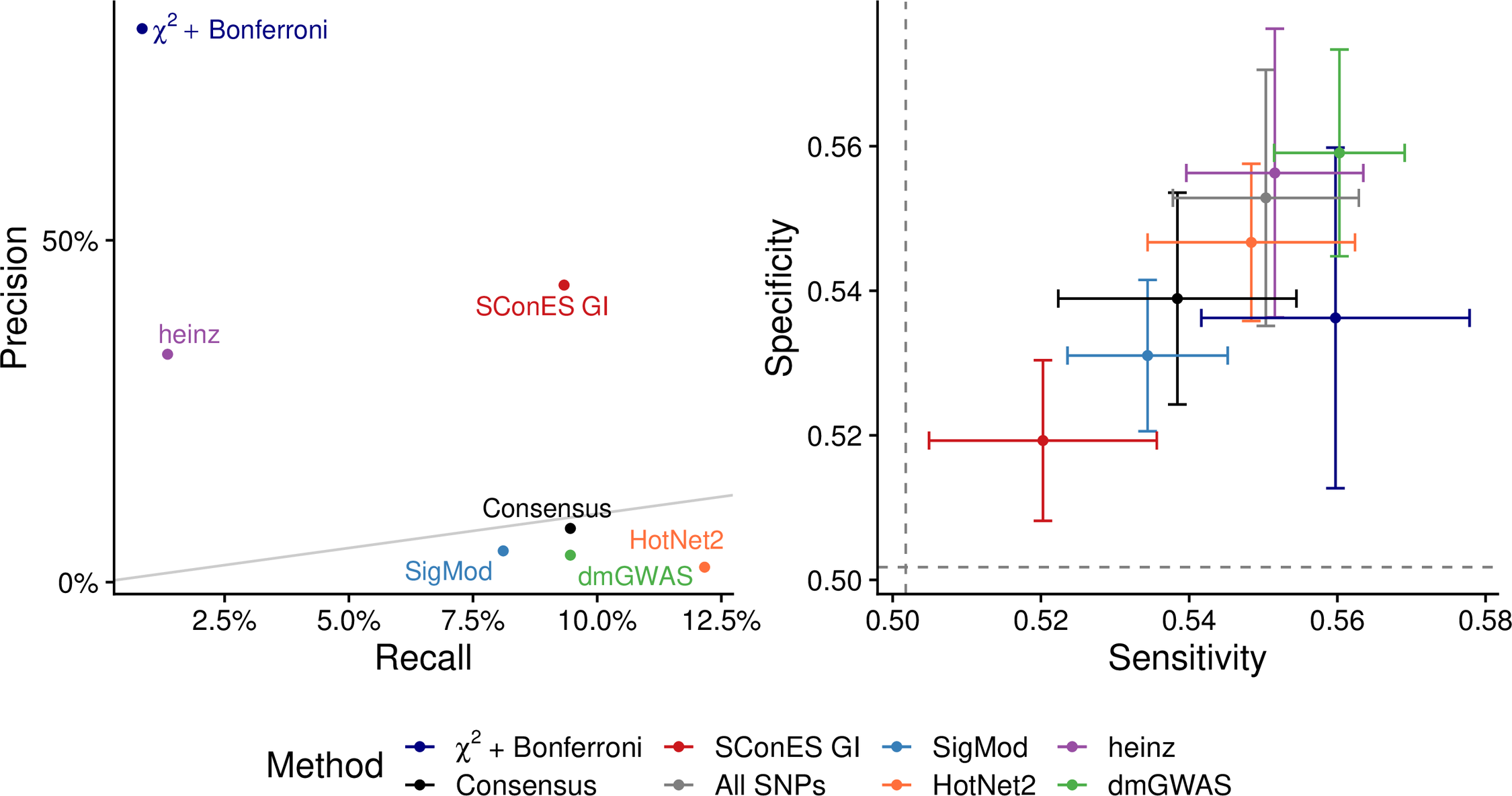

Supplement: S4 Fig — (A) Precision and recall of the evaluated methods with respect to Bonferroni-significant SNPs/genes in BCAC. For reference, we added a gray line with a slope of 1. (B) Sensitivity and specificity on the test set of the L1-penalized logistic regression trained on the features selected by each of the methods. The performance of the classifier trained on all SNPs is also displayed. Points are the average over the 5 runs; the error bars represent the standard error of the mean. (TIF) [file pcbi.1008819.s011.tif]

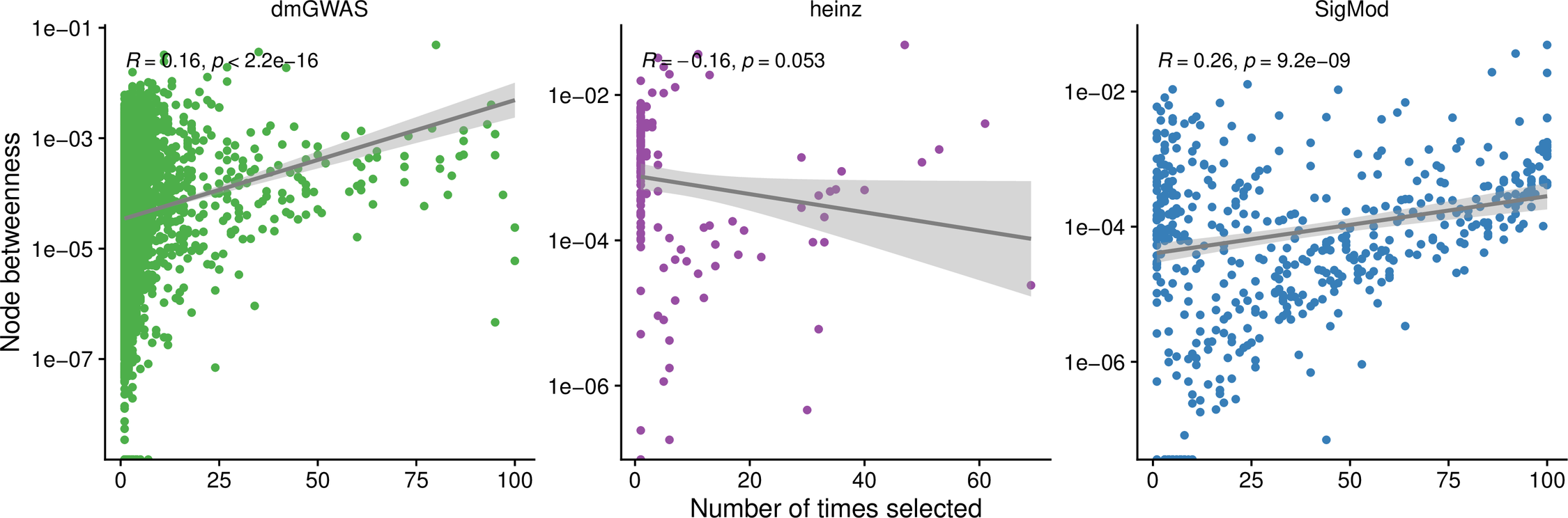

Supplement: S5 Fig — This figure is equivalent to Fig 4B, split by method. (TIF) [file pcbi.1008819.s012.tif]

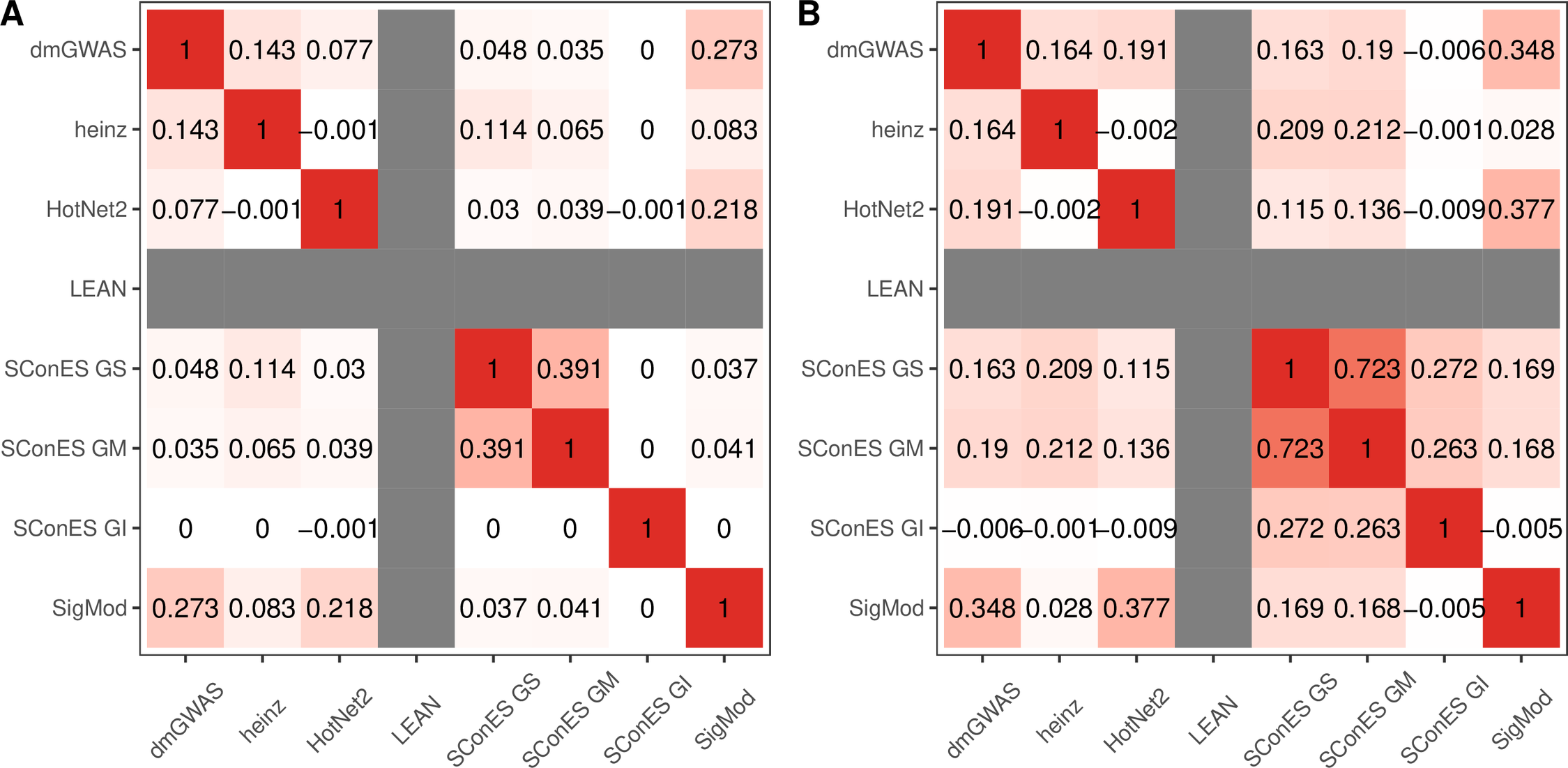

Supplement: S6 Fig — (A) Correlation between selected SNPs. (B) Correlation between selected genes. In general, the solutions display a very low overlap. (TIF) [file pcbi.1008819.s013.tif]

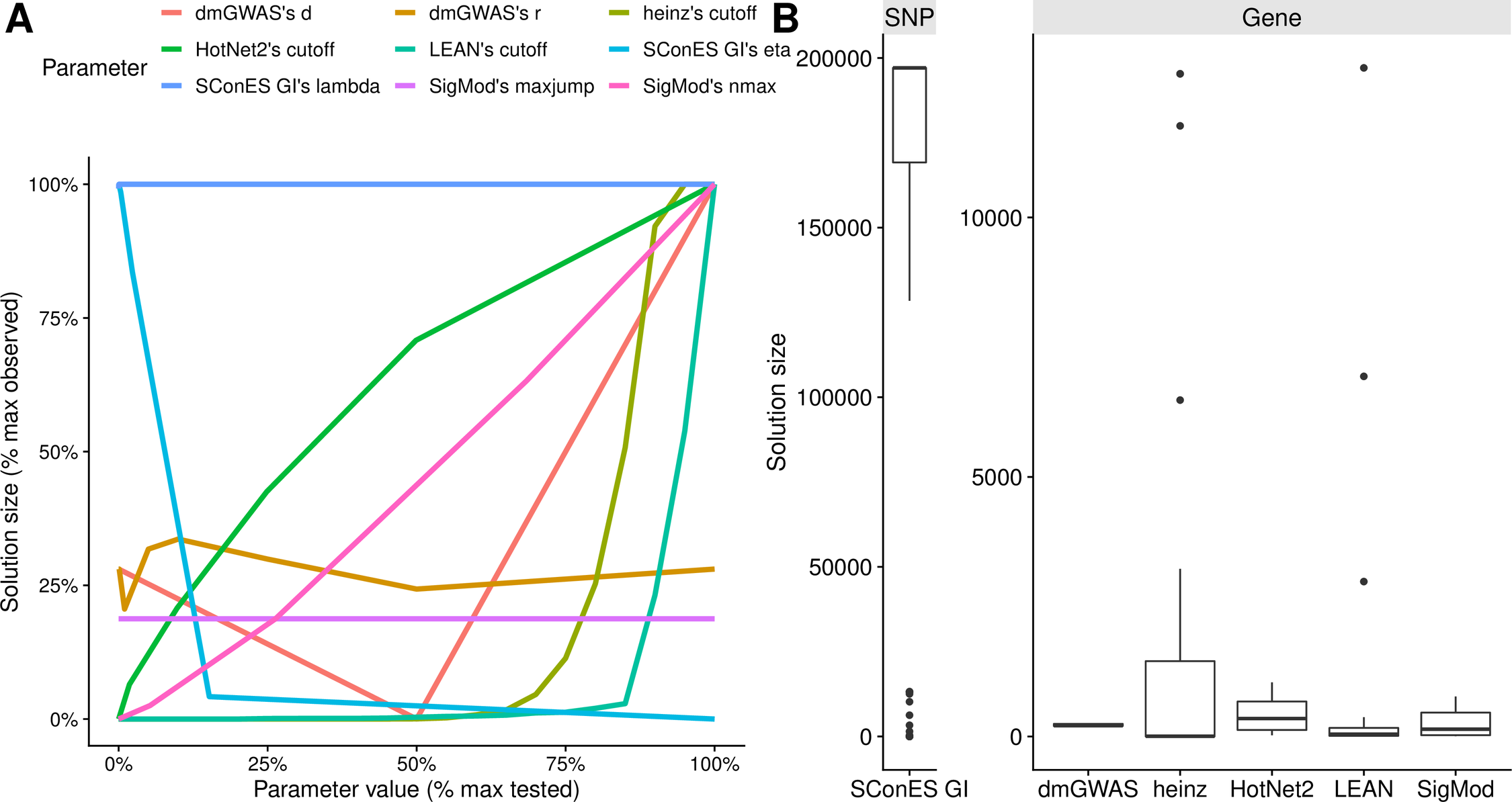

Supplement: S7 Fig — (A) Size of the solution produced by different parameter values, expressed as a percentage of the maximum solution size for the method, or the highest tested value for the parameter, respectively. The size of the solution is the median among all the solution sizes for the same parameter. (B) Boxplot of the solution sizes of the methods under the explored parameters (Section 2.3.4). (TIF) [file pcbi.1008819.s014.tif]

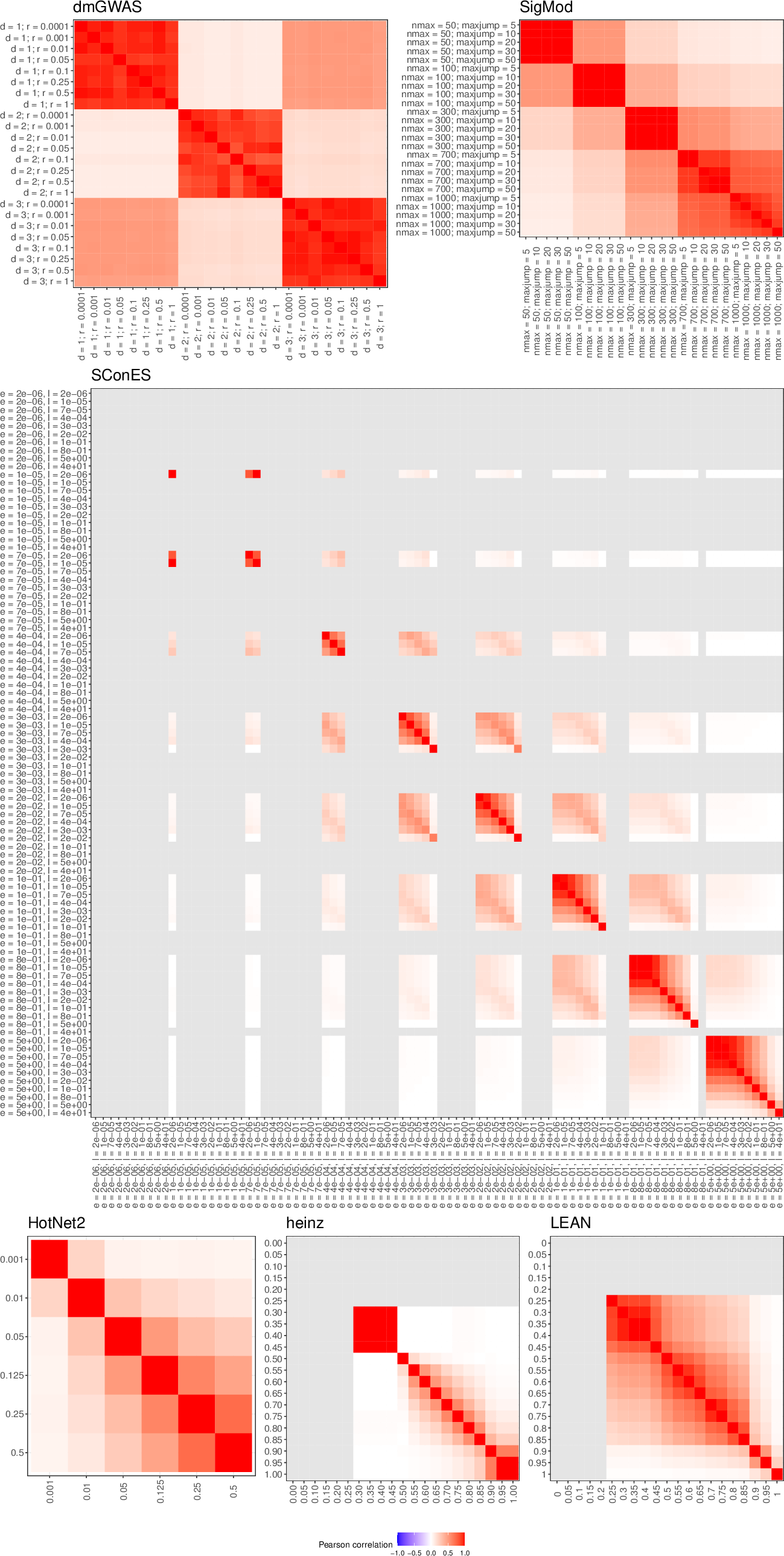

Supplement: S8 Fig — Grey tiles represent the cases where we could not compute the Pearson correlation because the two vectors were either all ones (all genes/SNPs were selected) or zeros (no genes/SNPs were selected). (TIF) [file pcbi.1008819.s015.tif]

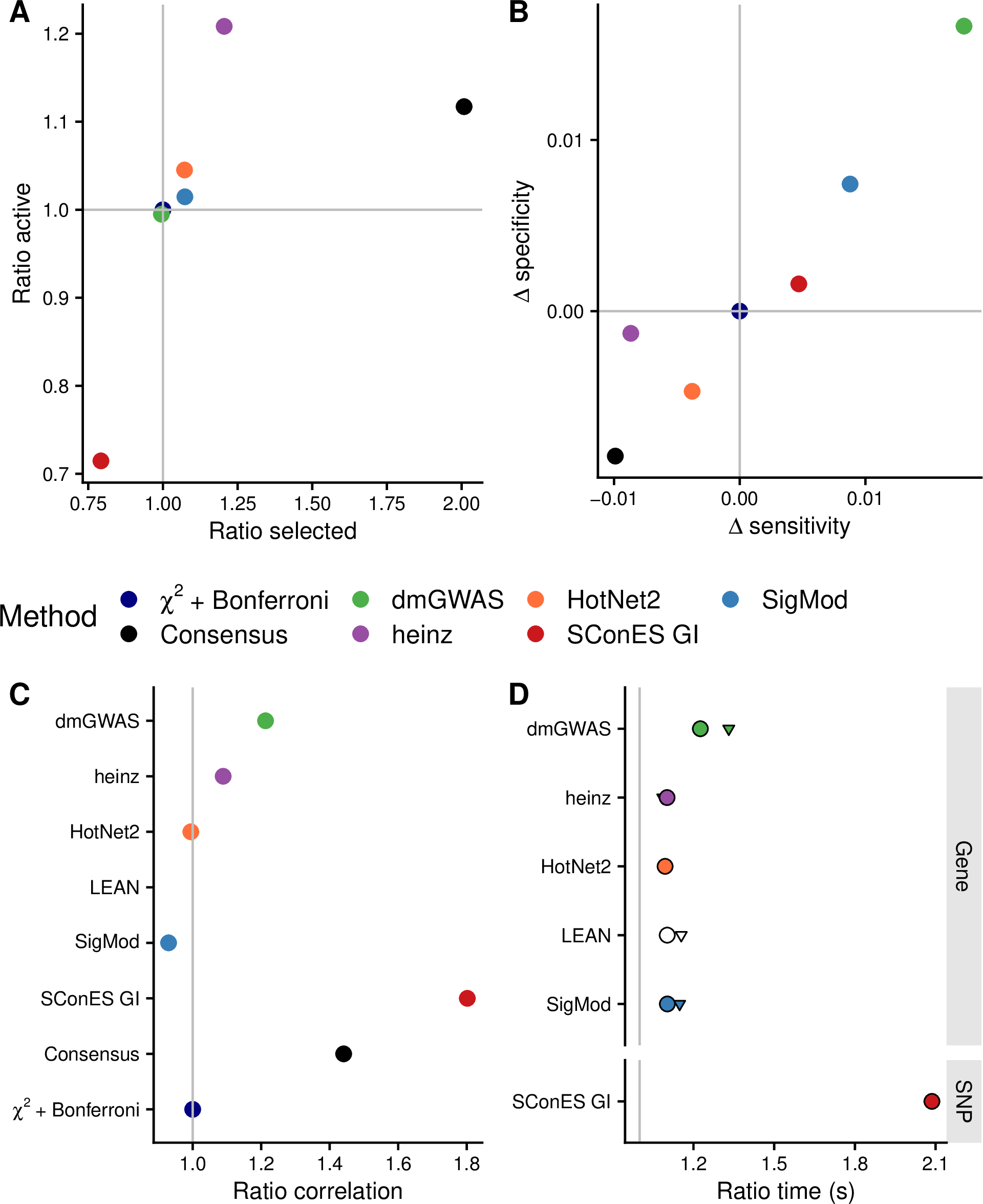

Supplement: S9 Fig — Grey lines represent no change in the statistic between the benchmarks (1 for ratios mean(HT) / mean(HT + LC), 0 for differences mean(HT)—mean(HT + LC)). (A) Ratios of the selected features between both benchmarks and of the active set (Section 2.5.2). (B) Shifts in sensitivity and specificity. (C) Shift in Pearson correlation between benchmarks. (D) Ratio between the runtimes of the benchmarks. For gene-based methods, inverted triangles represent the ratio of runtimes of the algorithms themselves, and circles the total time, which includes the algorithm themselves and the additional 119 980 seconds (1 day and 9.33 hours) that VEGAS2 took on average to compute the gene scores from SNP summary statistics. In general, adding additional interactions slightly improved the stability of the solution. However, it increased the solution size and the required runtime, and had mixed effects on the sensitivity and specificity. (TIF) [file pcbi.1008819.s016.tif]

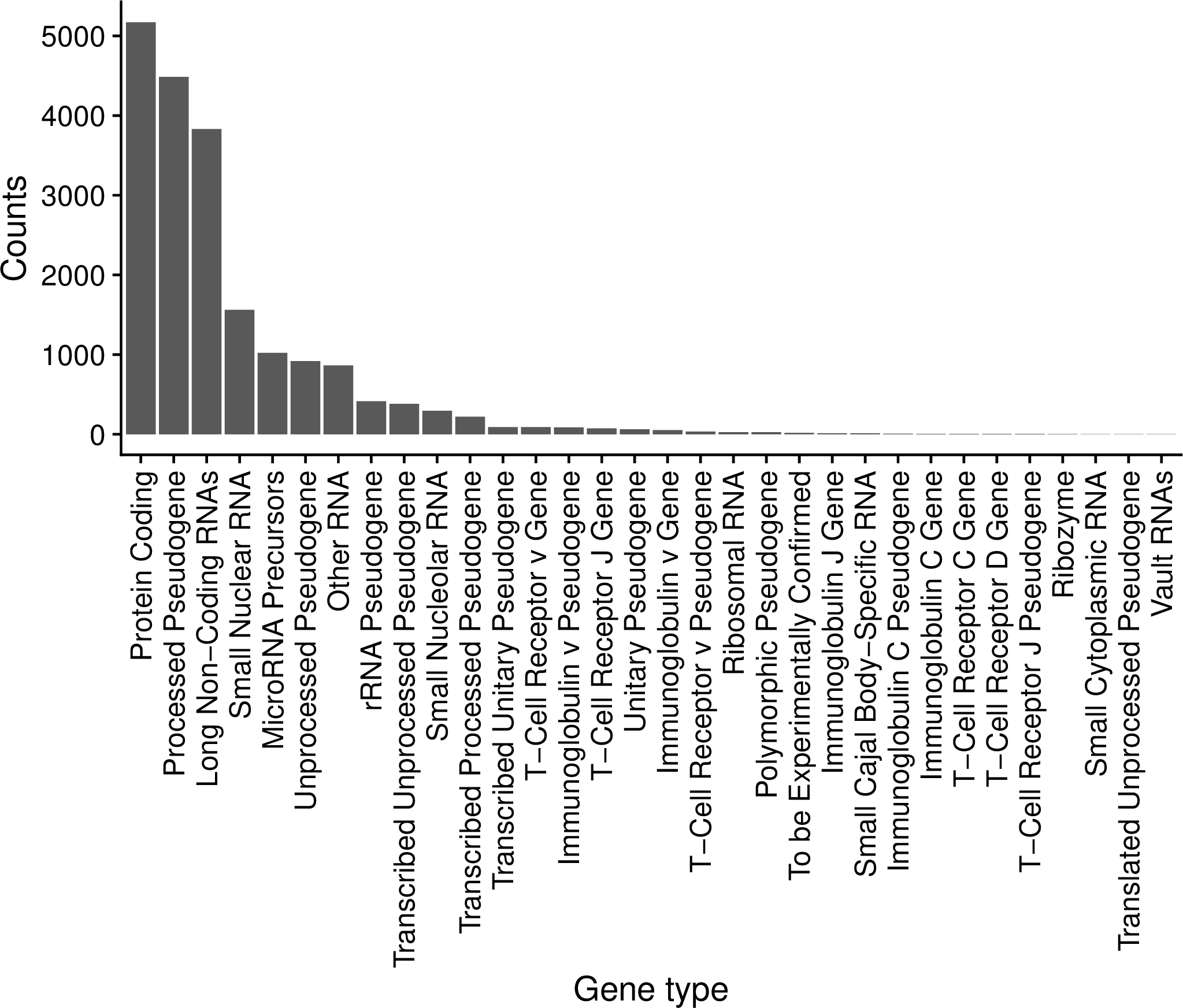

Supplement: S10 Fig — (TIF) [file pcbi.1008819.s017.tif]
