# Supplementary material for: Boosting GWAS using biological networks: A study on susceptibility to familial breast cancer
Source: PLoS Comput Biol. 2021 Mar 18;17(3):e1008819. doi: 10.1371/journal.pcbi.1008819 (PMC8009366; doi:10.1371/journal.pcbi.1008819)
Supplement: Attachment — Submitted filename: GENESIS Answer to reviewers.pdf [file pcbi.1008819.s018.pdf]

Dear Dr. Roy, dear reviewers,

We thank you for your consideration, attention to detail, and positive feedback on our manuscript. We have read and discussed the comments you raised with great interest and tried to address them in detail below (responses in blue). We believe this peer-review step has substantially improved the quality and comprehensiveness of the manuscript. We attach to this letter a final version of the article, and a marked version with all the changes.

We summarize here our main changes to the manuscript:

- We changed the title to one that better reflects the goals and achievements of this project: “Biological networks and GWAS: comparing and combining network methods to uncover the genetics of familial breast cancer susceptibility in the GENESIS study”. We also rewrote the Abstract.
- We added two important sets of experiments. One is on random rewiring of the network, in which we explore the output of the different methods when the biological meaning of the edges is lost, while the global topology is preserved. The other one is an exploration of the parameter space of the explored methods.
    - We have added a new panel 3B, including a ROC curve of the methods. Additionally, S7 and S8 Fig. which shows the size of the solutions obtained under different parameters, and the Pearson correlations between them.
    - We have added a new panel 4B, displaying the role that centrality plays in network methods. Also, S6 Fig. which splits that panel by method.
- A common critique was the deficiencies with regards to how we present the methods. Due to the methodological nature of this paper, we believe the paper will gain in clarity if we move the Methods section before the Result section. Since latexdiff makes it impossible to track the small improvements in Methods after such reordering, we submit the latexdiff version with the sections in the original order.
- Reorganization and re-ordering of the figures to better fit the flow of the text. Specifically:
    - We moved panels 1C and 3B to a new S6 Fig.
    - We have re-ordered the panels in Figure 1 according to their order of apparition in the text.
    - We merged figure 2 and supplementary figure 4, and figure 5 and supplementary figure 6. Additionally, we added Manhattan plots to both of them to display the statistical association of the selected genes.
- We provided the results of pathway enrichment analysis as supplementary files.
- We made an effort to make the text clearer and easier to read.
- We shifted the template to PLoS’.

Reviewer #1: This manuscript takes a systems biology approach to analyze the GENESIS data set of breast cancer GWAS. Overall, six network-based approaches were compared. A consensus network was created by taking all nodes identified by at least 2 network algorithms.

With so many competing computational methods that seek to solve the same problem, comparing the approaches on the same data set provides a helpful perspective. However, the manuscript does not provide an in depth comparison of the methodologies, does not perform parameter optimization for each methodology, and does not provide comprehensive documentation of the results.

Major revisions:

* Overall, the research question and gap that this manuscript seeks to fill is not clearly articulated. No new novel computational methodologies are presented and the results read more as a case report/review article.

Indeed, we recognize that this paper is at the intersection of a review of the method, a biomarker discovery endeavor, and what we consider our main contribution, the consensus network. Along this article we discuss the different mathematical formulations of the methods and their impact on the outcome. Since we do not find a reason to prefer any of them, we propose applying all of them and building a consensus solution. And to adjust for instability, what we consider the main downside of these methods, we propose a stable consensus. We have made that clearer in the Abstract and the Introduction, and changed the Title to reflect that.

In addition, the results from each of the network approaches are presented, but no comparison or contrasts are fully discussed. This is a missed opportunity for understanding the advantages of disadvantages of the methodologies.

We disagree with that characterization. Indeed, we compare the sizes of the solutions, the specific genes they include and the pathways these are involved in, their centralities, and association scores with the breast cancer susceptibility. Moreover, we frame these differences within the specificities of each method, to provide the reader with an intuition about their logic.

However, we admit that there was room for improvement in the previous version of the manuscript. We have added two additional comparisons to the manuscript:

- Following your suggestion below, instead of reporting only on results obtained for default parameters of the network-guided methods, we are now also reporting how these parameters influence solution size and the selected genes (Sections “Parameter space” and “Different parameters produce similarly-sized solutions”). With caveats, this helps at characterizing the potential of methods at recovering biomarkers.
- Based on a suggestion from Reviewer 3, we also ran the methods under a random rewiring of the network, keeping degree constant (Section “Network rewirings”). This sheds light on the role of node centrality.

* The introduction was non-specific. If the goal of the manuscript is to highlight the challenges of identifying SNPs in breast cancer, then the introduction should highlight the prior studies that

have attempted to identify SNPs. If the goal of the manuscript is to focus on analytical methods, then more discussion of other approaches for analyzing GWAS data is necessary.

We agree that what we consider to be our main contribution/message was unclear. We are proposing to build a consensus network, and the aim of our manuscript is to motivate this proposition on a concrete example. Note also that not all the methods we used had been applied with GWAS data before (some used gene expression data). Despite not focusing on BRCA, we analyze both GENESIS and BCAC and test multiple methods. Then, we propose to combine the methods, which is not something we often see. In addition, we provide the means for researchers to apply them to their data. We changed the Title accordingly to "Biological networks and GWAS: comparing and combining network methods to uncover the genetics of familial breast cancer susceptibility in the GENESIS study".

* The methods and results lacked reason and justification for decisions on parameter values. Selection of parameters could impact the final results substantially. Since the goal of the manuscript is to compare and contrast different network approaches, more exploration of the parameter space of each methodology is needed.

This is a good question in the context, which naturally arose during the project. Some methods provide guidance about how to set the parameters, specifically HotNet2 and SConES. LEAN only requires selecting a P-value cutoff on the results. Similarly, heinz requires selecting an FDR threshold. The User manual of DmGWAS v2 recommends using d = 2 and r = 0.1. SigMod's documentation suggests testing different parameters, specifically for *nmax* and *maxjump*; although it offers no guidance about which solutions are preferable. In the text, we discuss these parameters, and their impact on the final solution.

At multiple points in this project we discussed which parameters to use. If we had a complete set of breast cancer susceptibility genes, the approach would be straightforward: we could find the parameters that best recover it. In the absence of a gold standard, we could have looked for parameters that produce properties with desirable solutions. Yet, what those properties should be is in itself arguable (High association scores? High modularity? Highly central genes?), and might lead to circular reasonings where we choose the parameters that allow us to recover our preconceived ideas. The last option we considered is to use simulation studies. But how accurately simulate phenotypes from genetic data is in itself an open question. Again, we would likely overfit to the model and initial assumptions we chose.

With these considerations, we have explored the problem of parameter selection in the new version of the paper. You will find the details of the experimental setup in Section "Parameter space", and the results themselves in section "Different parameters produce similarly-sized solutions".

* It is unclear what information was provided to each of the network methods. Specifically, all but one network algorithm uses gene level values such as p-values for each gene. The

approach to merging SNP level data to gene level data for the network methods is not described.

We use VEGAS2 P-values. We clarified that in the text.

* The precision/recall of these methodologies compared to BCAC are underwhelming.

First, we would like to point out the shortcomings of using BCAC as a gold standard. BCAC itself does not explain the full heritability of breast cancer susceptibility. Hence, by definition, an ideal method that does find all true biomarkers will have a suboptimal precision on BCAC. Additionally, BCAC compiles multiple European populations. Hence, an ideal method that finds all true biomarkers in the French population might also show a suboptimal sensitivity. This is exacerbated by the predictions of the omnigenic model, which questions that a GWAS like BCAC can, indeed, explain the full heritability. We had a paragraph in Methods explaining the shortcomings of BCAC as a “gold standard”. To highlight these considerations to the readers, we have expanded it and moved it to the Discussion.

It is more preferable to have high precision or high recall?

With the caveats above in mind, we would argue that it depends:

- A high recall might be preferable in exploratory analyses in which the goal is to identify potentially causal genes in follow-up experiments. In that situation, we find it advantageous to have access to most biomarkers. What is more, we find it advantageous to include the biological context of the selected genes to better understand the function of the gene. Additionally, as the omnigenic model predicts, many genes involved in the disease are not expected to be found in GWAS experiments.
- A high precision might be preferable when a small set of highly informative biomarkers are sought. For instance, for diagnosis.

We have added these considerations to the Discussion.

* The pathway analysis methods results were under-reported. This analysis was performed for each network algorithm result and the consensus network. No full pathway analysis results are present in either the main text or supplemental document. The network algorithm results were deemed “similar”, but this cannot be confirmed.

You are absolutely right, thank you for noticing. Although we never mention that in the text, the full list was available at https://github.com/hclimente/genewa/blob/master/doc/consensus.ipynb. Our intention was not to overwhelm the reader with multiple lists of significant pathways, but interested readers could always access them. Additionally, another paper focussing on pathway analysis of the full GENESIS data has been written in parallel to this study, and submitted to other venue. However, we see now that that was not the best course of action, and have now added all the performed pathway analyses as supplementary tables 3-6, and referenced them on the main text.

Moreover, the results were manually curated with no criteria for such curation.

By *curation* we meant that we highlighted in the text only some of the pathways. Since we provide the full lists now, we have removed that confusing wording.

Were pathways selected because they were supported by literature or because they represented common themes?

Since enumerating all the enriched pathways in the main text would be cumbersome, we highlighted the pathways that best represented a particular solution. In our view, those were non-redundant pathways that contained different sets of genes. Only then did we search for support in the literature and agreement with the solution of the other methods.

* The consensus was determined by taking the set of nodes that were present in at least two of the six methods. How would the results vary if a different number of method identification were used?

That is an interesting question that deserves some comment. We have added the following sentence to the results: “Indeed, out of the 668 genes that are selected by at least one method, only 93 are selected by at least two, 20 by three, and none by four or more. Encouragingly, the more methods selected a gene, the higher its association score to the phenotype (Fig 1B), a relationship that plateaus at 2.”

Also, there is no description how the edges were identified in the consensus network.

We added the following sentence: “Any edge between the selected genes in the PPIN was included.”.

Minor revisions:
* The figures are referenced out of order.

That is, indeed, an oversight on our part that the other reviewers have also pointed out. You will find in our summary above the comprehensive list of figure changes to address this.

* “Heinz produced the smallest solutions, with an average of 182 selected SNPs”. It is unclear how the gene-level network methods select SNPs.

We have added the section “Comparing SNP-methods to gene-methods, and vice versa” that explicits this mapping and referenced it here.

* How are Pearson’s correlation of a solution set computed.

We realize now that this computation is not self-evident. It involves creating vectors of size equal to the number of features (in this case, the number of genes in the PPIN, or SNPs in the array). For a particular run, position *i* will contain a 0 if the *i*-th feature was not selected, and a 1 if it was. Then, the Pearson correlation between the two vectors of two different runs can be computed. We explicit this in Section “Properties of the solution”.

* What were the computing resources available on the machine used for testing the algorithm runtime?

We apologize for the oversight. We added a new section, entitled “Computational resources”, containing this information.

Reviewer #2: The paper entitled “Comparing and combining network-guided biomarker discovery to discover genetic susceptibility mechanisms to breast cancer on the GENESIS study” proposes the implementation and comparison of different algorithms to identify subnetworks of interest from the integration of network and GWAS data. These applications are done in the particular context of the GENESIS study, a french GWAS of patients without BRCA1/2 pathogenic variants.
Overall, the approach and results are interesting, in particular the comparison of the outputs of different algorithms used to leverage network data are of interest to the community. However, despite this relevant content, the paper suffers from style and organisation weaknesses making it difficult to follow.

General comments:
Take care of using the same word(s) when referring to a unique concept. For example, “stable consensus” and “stable consensus subnetwork” are both used, or “molecules”, “biomolecules” and “genes”. Other similar examples of unnecessary use of synonyms are “PPIN”, “protein-protein interaction network” “gene-gene interactions” and “network”, and “solution network”, “causal network”, “causal subnetwork”, ”subnetworks”, “susceptibility network”. Similarly, please use the same formulation to refer to the 3 breast cancer datasets along the text. Overall, the manuscript would highly benefit from a simplification of the text. The style is sometimes unnecessarily complicated, please simplify.

Indeed, we agree that there was great room for simplification. We did so by making the following replacements:

- *Benchmark* for the cross-validation comparison;
- *PPIN* for most instances of a gene-based network;
- *Network methods* for references to the studied methods;
- *Solution* for the subnetwork solution of the different network methods, including *Consensus solution*.

Also, we made many smaller changes to make the text more accessible.

The reference numbering does not start at [1].

Thanks, we fixed it.

The figure numbering does not start at 1 in the text, and does not follow the order of quotation of the figures. For instance, in the results section you start referring to your supplementary materials with the Supplementary Figure 2A whereas you haven't mentioned the Supplementary Figure 1. Figure 3B and 1B are quoted before Figure 1A, Figure 1C is referred after Figure2…

Thanks for these observations, we have reorganized the figures to be enumerated in a consistent order. You will find the whole list at the beginning of this letter.

Overall, the referencing organisation of the paper lacks clarity. Aside from the reference and figure numbering, the method section is not quoted when needed. For instance, the results start by quoting Section 4.3.1 in the first sentence, but it's not mentioned that this corresponds to the method section, and the previous sections of the method section, explaining the first parts of the analyses, are not quoted in the results. Please use consistent and logical organisation, in particular to quote the method section.

Thanks for pointing this out. In the new version of the manuscript, we have made an effort to quote the relevant parts of the Results and the Methods where they are needed.

Several acronyms are used for the first time without a definition (e.g. PPIN and BH).

Thanks, we fixed those.

The network figures 2 and 5 could be improved for readability and for information content. Please consider improving the visibility of the nodes and edges, removing the lines pointing at the gene labels because it looks like additional edges. In addition, having only the names of some of the nodes lowers the interest of the figures. The networks are not so big, and a software such as cytoscape should allow to display both node pie charts and all node labels on the figure.

We agree with this suggestion. We simplified the networks in figures 2 and 5. Also, we added supplementary figures 4 and 6 respectively as panels, which show the name of every gene in the network. Lastly, we added an additional panel containing the Manhattan plot using VEGAS2 P-value, with the genes in the solution highlighted.

Add numbers to all the equations, only one equation is currently numbered

In the previous version, we were numbering only equations that we referred to in the text. We agree that numbering all equations might be advantageous for readers, and changed the manuscript accordingly.

Be consistent with the use of tenses. For instance, in section 4.3.2 you speak in present tense, but in section 4.3.3 you speak in past tense.

Thanks, we went over the whole manuscript to be consistent in our tenses.

The global organisation of the figures might be redesigned, as it does not seem logical nor aligned with the flow in the text. For instance, figure 1D could go with figure 2, Figure 1C could go with figure 3 … The current flow of the text is scattered in the different figures, please consider redesigning the figures to follow the main ideas and sections.

That is, indeed, an oversight on our part that the other reviewers have also pointed out. You will find in our summary above the comprehensive list of figure changes.

Many formulations and sentences could be more specific, such as “we show a general alteration of the neighborhood…”, “significantly large overlap...”, “larger subnetworks...”, “it is located near...”.

Thanks, we added numbers to support these statements.

Detailed comments on the text

Abstract

The sentence “By trading statistical stringency for biological meaningfulness, most network methods give more compelling results than standard SNP- and gene-level analyses, recovering causal subnetworks tightly related to cancer susceptibility.” is not clear, consider rephrasing. In addition, do the methods really trade statistical stringency? They just use other types of statistical tests, which are also selected for significance.

We think you are correct. When we wrote stringency, we meant type I error: network methods are less worried about introducing a gene with a very low score, if it allows them to connect to high scoring genes. Indeed, some network methods do perform a statistical test on their solutions. We have removed this sentence from the abstract.

In the sentence “Yet, network methods are notably unstable, producing different results when the input data changes slightly.”, what do you mean by “slightly”? Does it apply to the network, the GWAS results or to both? Is this sentence a result of the study or a statement from the background knowledge?

This sentence referred to the instability of the network methods that we show in our experiments using a Pearson correlation. Specifically, the correlation between solutions on different subsets is 0.45 on average for network methods, and 0.55 simply applying a Bonferroni correction.

“More central positions in the network than average” => than average what ?

Than the median gene in the PPIN. We have replaced that expression by “than most genes”.

Author summary
“pipeline” is not clearly defined

We have replaced that sentence by “We provide ready-to-use implementations of all the examined methods.”.

Introduction
Syntax: “Bonferroni correction is overly conservative when the statistical tests are correlated, as it* is the case in GWAS”.

Thanks, we fixed this typo.

List a couple of such properties in the sentence “In fact, an examination of biological networks shows that disease genes have differential properties.”.

Good suggestion, we cited some such properties.

The sentence “Network-based biomarker discovery methods exploit this relatedness to identify disease genes on GWAS data” is not clear, consider rephrasing. What does “relatedness” mean here?

We replaced that construction by “Network-based discovery methods exploit the differential properties described above to identify disease genes on GWAS data”.

“Such is the extreme case of LEAN [25], which focuses on “star” subnetworks, i.e. instances were both a gene and its direct interactors are associated with the disease.” -> does this apply to all the neighbors?

No, LEAN conducts a statistical test on each star subnetwork to verify if the P-values are lower than expected for similarly-sized networks. The previous formulation was confusing, so we replaced it by “Such is the extreme case of LEAN [25], which focuses on “star” subnetworks, i.e., instances were both a gene and its direct interactors are, on average, associated with the disease.”

The sentence “While all of them capture susceptibility mechanisms resembling that postulated by the omnigenic model, they do so in different ways, and provide a representative view of the field.” is not clear, consider rephrasing.

We have simplified that sentence to “They use different interpretations of the omnigenic model, and provide a representative view of the field.”.

The introduction states a lot of concepts that could be defined more precisely to avoid confusion. For instance, a proper definition of biomarker could help as it can be seen as all the nodes present in a discovered subnetwork, or only the nodes with a significant p-value, or as cancer drivers, for instance. These concepts of significant genes, cancer drivers, biomarkers … are used without proper definitions.

We noticed that the manuscript could be improved by removing some of those terms, or replacing them by easier to understand, and more accurate, terms. We replaced “biomarkers” by “susceptibility genes” when necessary, including in the title. And since we are working on cancer, but this is not a cancer driver discovery project, mentioning them might be confusing for the readers. Hence, we have removed them from the introduction. With regards to the term “significant genes”, we have specified how we define significance in each case, and in which dataset.

Results
Section 2.1
“Moreover, and in opposition to what would be expected under the omnigenic model … are not interconnected in the PPIN” => I’m not sure the omnigenic model would lead to this statement, in particular as it is defined in the introduction. This is more a statement of network biology.

We would argue it is both about systems biology and the omnigenic model. We mention in the introduction that the omnigenic model predicts that “genes are very functionally interrelated and influence each other's behavior, which allows alterations in most genes to impact the subset of “core”' genes directly involved in the disease's mechanism.”. In other words, the susceptibility genes are tightly connected to each other.

The sentence “In addition, the classification performance of the method is very low, and L1-penalized methods select only one of several correlated variables and are prone to instability, which further complicates interpretation.” is not clear, consider rephrasing.

Thanks for this observation. We have reduced that sentence to the main problem with this approach “Moreover, the classification performance of the model is low (sensitivity = 55%, specificity = 55%, Section 4.5.2).”.

In the sentence “This motivates exploring network methods, which trade statistical significance for biological relevance to find susceptibility subnetworks.”, what does “susceptibility

subnetworks" mean? Please consider this comment in light of the general comment about the use of synonyms to refer to the same concept.

Indeed. As pointed out in our previous answer, we have made an effort to use consistent words for the same concept. In this case, we have replaced that by 'susceptibility genes'.

In addition, there is a discrepancy in the organisation of the arguments in this section, because it states "the genes to which these SNPs map to are not interconnected in the PPIN" and "This motivates exploring network methods".

We understand the concern. We hoped the reasoning would flow as follows: a PPIN-agnostic approach does not select genes that are connected in the PPIN; but, we have reasons to believe (omnigenic model, system biology) they should be; hence, we should explore methods that impose that restriction on the solution. We have changed the text accordingly to reflect that intermediary step, by adding the sentence "Moreover, and in opposition to what would be expected under the omnigenic model, the genes to which these SNPs map to (Section 4.3.5) are not interconnected in the protein-protein interaction network (PPIN, Section 4.3.2).".

Consider rephrasing the sentence "In fact, such methods provided comparably (poor) classification performance to L1-penalized logistic regression (Figure 3B), while providing more interpretable solutions." to state more precisely the underlying idea, as bad results could be considered as bad results and thereby cannot really be interpreted.

That sentence, in the absence of proper discussion, raises more questions that it solves. We decided to remove it since we discuss this point in a more appropriate context later in the manuscript.

Section 2.2
It's not clear how the overlap is calculated with a Pearson correlation.

Quoting our answer to the same concern bi Reviewer #1: "We realize now that this computation is not self-evident. It involves creating vectors of size equal to the number of features (in this case, the number of genes in the PPIN, or SNPs in the array). For a particular run, position *i* will contain a 0 if the *i*-th feature was not selected, and a 1 if it was. Then, the Pearson correlation between the two vectors of two different runs can be computed. We explicit this in Section "Properties of the solution"."

Why not use Jaccard similarity or adjusted Rand Index?

We refer to the argument made by Nogueira and Brown (2016). In summary, they enumerated four desirable properties that a stability measure should have:

1. It is fully defined.
2. It is bounded below and above.
3. The highest value is achieved if and only if vectors are equal.
4. It corrects for chance.

Pearson correlation fits these four criteria: it produces a value regardless of how many features are selected; it's bounded between -1 and +1; two equal vectors produce a value of 1; and the expected value from two random vectors is 0.

Regarding those two measures specifically, they do not meet all those four criteria. For instance, Nogueira and Brown show how the Jaccard similarity monotonically grows as the size of the two random vectors increase. Nogueira and Brown do not include the adjusted Rand Index in their comparison. Indeed, the Rand Index is not an appropriate measure of similarity between sets of selected genes, because it is meant to compare *unlabeled* clusters (or, equivalently *unordered* partitions); whereas a feature selection procedure partitions the features in two *labeled* sets, the set of selected features and the set of unselected features. In other words, if a first feature selection procedure selects a subset A of the features, and a second feature selection procedure selects exactly the complementary of A, their Rand Index would be that of a perfect match (equal to 1), whereas these two procedures yield entirely opposite results and should get a similarity score of 0.

LEAN is described as not retrieving "networks" in the text, and "genes" in the figure legend.

Thanks, we fixed this inconsistency.

"While SConES GI failed to recover any protein coding gene..." -> is the goal to retrieve protein coding genes?

Indeed, retrieving protein coding genes is not the goal. We replaced "failed to" by "did not".

Figure 1.
(C) Put the names of the methods as legend, to improve readability. This could also be done for the similar pictures in Figure 3.

We have done so in both Figure 3, and Figure S5 (previously Figure 1C).

(D) The consensus network is the same as in Figure 2. Please consider fusing these Figures.

Since we do not discuss the consensus until later in the text, we have removed it from this figure.

Bonferroni thresholds mentioned here in the legend but not in the method section

We have created a new section in the methods, “SNP- and gene-based GWAS”, where we explain how we perform a “conventional GWAS” and, specifically, describe the Bonferroni correction. This section replaces and extends the section entitled “SNP and gene association” in the previous version of the manuscript. Importantly, we created an extra new section “Comparing SNP-methods to gene-methods, and vice versa”, containing the parts of the old section that were not included in the new section.

Table 1.
Add the number of connected components.

Thanks, we added that column.

In the sentence “Lastly, a pathway enrichment analysis (Section 4.4) also showed similarities and differences in the underlying mechanisms.”, are you talking about all the methods or only about HotNet2 results? Underlying mechanisms of what? Not clear, consider rephrasing.

Indeed. We have replaced the sentence above by “Lastly, a pathway enrichment analysis (Section 4.4) also showed similarities and differences between the solutions of the different methods”.

Section 2.3
“Despite the heterogeneity of the solutions, their shared properties suggest that each method captures different aspects” => this seems self-contradictory

We agree. We have replaced it by “Despite their shared properties, the differences between the solutions of the different methods suggest that each of them captures different aspects of cancer susceptibility.”.

In the sentence “we built a consensus subnetwork that captures the mechanisms most shared among the solution subnetworks (Section 4.3.4).”, how do you define a mechanism?

We notice that mechanism is an ambiguous term. In this case, it referred to the genes and, as we develop later, pathways. To avoid confusion, we have replaced it by “we built a consensus solution that captured the genes shared among at least two solutions (Section 4.3.3).”

What is the overlap between at least 3 methods? And between all the methods?

That is a relevant question, that other reviewers have also inquired about. We added the following lines: “Indeed, out of the 668 genes that were selected by at least one method, only 93 were selected by at least two, 20 by three, and none by four or more. Encouragingly, the more methods selected a gene, the higher its association score to the phenotype (Fig 1B), a relationship that plateaus at 2.”.

Which tool did you use to do the pathway enrichment analysis?

We used ReactomePA's *enrichPathway()* function. We mentioned this function to the methods.

Why do you refer to VEGAS2 p-values in this section and BH or Bonferonni in the previous ones? Do all these different words refer to different experiments?

When we refer to gene P-values, we always refer to VEGAS2 P-values. The BH P-values refer to other P-values (LEAN and pathway enrichment analyses). With respect to Bonferroni, it is indeed a cutoff we apply on VEGAS2 gene P-values (as well as SNP P-values). To avoid confusion, we have changed the text to consistently mention gene P-values as "VEGAS2 P-values".

"The opposite" => to what?

We are referring to the largest connected component. We replaced "The opposite..." by "In the former we find…", which links the ideas better.

"This suggests that they were selected" => "they" refer to what?

Highly central genes with low P-values. We clarified it in the text.

"Despite its lack of association … its neighbors in the consensus …" => and in the PPIN?

Once a method selected *COPS5*, it would also tend to include most of its low P-value interactors. Indeed, while the median P-value of the neighbors in the consensus is 0.006, that of those neighbors in the PPIN that were not included in the consensus is 0.434.

Section 2.4
This section starts with the precise description of the BCAC dataset, which has been mentioned already, for instance in the legend of Figure 2. Please consider a reorganisation to give the information when needed first.

We took two measures to easen this. First, we moved the Materials and methods section before results. Second, we quote the relevant section of the methods the first time we mention BCAC.

Typo "...at which most network methods operate."

Thanks, we fixed the typo.

What is the correlation between BCAC and GENESIS at the gene level?

Although it did not make it to the final version of the article, we published this analysis [on our Github repository](#):

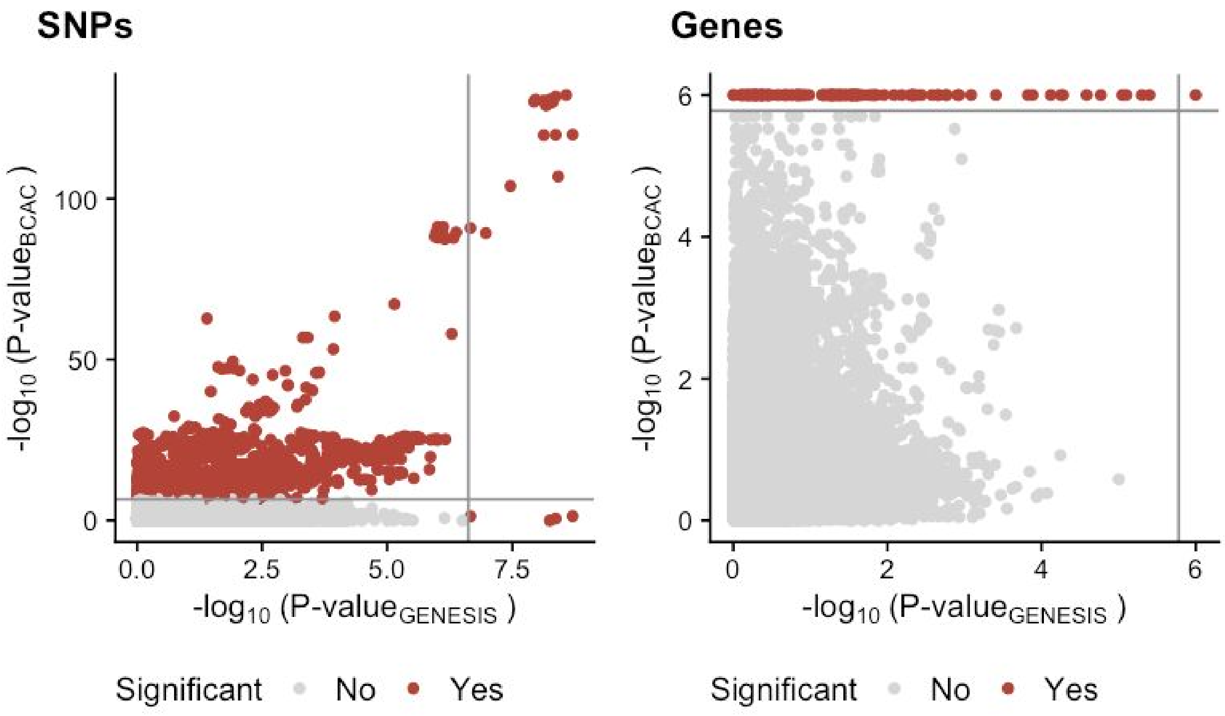

The lines represent the Bonferroni thresholds. These thresholds create 4 spaces at the SNP level, and another 4 at the gene level:

	SNP		Genes	
	Non-significant GENESIS	Significant GENESIS	Significant BCAC	Significant GENESIS
Non-significant BCAC	207,809	4	29,857	0
Significant BCAC	2,008	17	128	1

When we conduct a Fisher's exact test on these two contingency tables to study the overlap. We observe that the overlap is significant in both cases (P-value < 2.2e-16 at the SNP level, P-value = 0.004 at the gene level).

Section 2.5
Add the characteristics of the computer where you run the experiments in the method section.

We apologize for this oversight. We added a new section in methods, “Computational resources”, that provides the specifications of our computing cluster.

Why “we do not expect to separate cases from controls well using exclusively genetic data”?

Generally speaking, predicting complex traits is hard. It is more so in the case of phenotypes with incomplete penetrance, like cancer. Even considering all the known environmental risk factors, predicting trait disease based on genetic component is still difficult. In the genetics community, this is known as the “missing heritability” problem. Some of the possible explanations are that the phenotype is highly dependent on rare variants which we are not surveying in GWAS; that the small effects of the variants are too small for the statistical power of GWAS; or that we are not accounting appropriately for genetic interactions. We have added a reference to the manuscript to support this claim.

Section 2.6
The sentence “This is due to the particularities of each method, and directly or indirectly provides information about the dataset.” is unclear, consider rephrasing.

We agree. We replaced that sentence by “This is due to the fact that each of them models the problem differently. Importantly, understanding which assumptions they make allows to understand the results more in depth.”.

Figure 3.
Add the legend in an independent box to avoid overlapping text in (A) and (B).

Thanks, the new figure is, indeed, less cluttered.

The methods in (C) and (D) are listed in different order.

Thanks, the methods are listed in the same order now. We also changed the order in Supplementary Figure 8.

(D) The inverted triangle of SConES GI is missing (or not visible).

The inverted triangle represents the time taken by VEGAS2 to compute gene scores. Since SConES works directly on SNP scores, that step is omitted. We added a legend to the figure to clarify this.

For consistency, however, we have replaced SConES GI’s circles by triangles.

Why is there a blank space between the legend “Gene” and “SNP”?

We found it helpful to separate visually Gene-based methods from SNP-based methods.

What “conventional GWAS” refers to? What is the difference with “Genesis-significant” used in Figure 1?

‘Conventional GWAS’ refers to the following procedure: given a GWAS dataset (or subsample of one), a χ^2 test is performed, followed by a Bonferroni correction to obtain significant SNPs. ‘GENESIS-significant’ involves applying ‘Conventional GWAS’ to the whole GENESIS dataset. By subsampling the data and applying ‘Conventional GWAS’ (for instance, the ones in Fig 4), we might produce a set of significant SNPs that is different from ‘GENESIS-significant’.

Upon reflection, we agree that this terminology is confusing. Hence, we replaced both terms by the more explicit ‘χ^2 + Bonferroni’. This applies to Figures 1 and 3, as well as Figs S5, S9 and S10.

What is a solution “used”, do you mean produced?

Yes, thanks, we fixed the typo.

What do you mean by “active set”?

The active set is the set of SNPs with a coefficient different from 0 in the penalized logistic regression. We cited the section of the methods where it is explained.

Which lines represent the standard error of the mean?

Indeed, “lines” is an ambiguous term in Figure 3. It refers to the error bars. We specified that.

Figure 4.
Why did you use a threshold of P-value < 0.1? Does this correspond to the GWAS p-values?

Yes, it corresponds to GWAS P-values. The threshold represented a way to roughly separate “low P-values” from “high P-values”. We have replaced that arbitrary threshold by a color gradient representing the -log10 of the P-values.

Figure 4C is not clearly explained.

We have simplified Figure 4C and adapted the text to better describe the underlying idea.

Figure 4B, why is this computed on BCAC and not on GENESIS data?

Because BCAC is more powered than GENESIS. Hence, with the caveats we mention in the Discussion and in our answer to Reviewer 1, we expect that it will be better at finding significantly associated genes. Since it might happen that a "good gene", which is significant in BCAC, is not significant in GENESIS, and yet, network methods will pick it because of its location in the PPIN.

Figure 4A, why not using a color scale to display the significant p-values, instead of significant/non-significant?

Thanks for this suggestion, the new figure is more informative.

Add the name of the dataset in the sentence "In this dataset, heinz's solution is very conservative...".

We have replaced that by "In the GENESIS dataset...". We have also specified the dataset in a few other instances that seemed ambiguous.

In the sentence "Due to this parsimonious and highly associated solution, it was the best method to stably select a set of biomarkers (Figure 3C).", what do you mean by "parsimonious"? Highly associated with what?

This is just a restatement of the previous sentence (small and highly associated on median). Hence, we rephrased that as "By repeatedly selecting this compact solution, heinz was the most stable method (Fig 3C).".

In the paragraph starting with the sentence "On the other end of the spectrum, dmGWAS, HotNet2, and SigMod produced large solutions.", the use of the terms such as "least associated" and "weakly associated" is not clear.

We have replaced "least associated" by "lowest scoring", and "weakly associated" by "low scoring".

The sentence "In fact, due to linkage disequilibrium, SConES favors such genes, as selecting SNPs in an LD-block which overlaps with a gene favors selecting the rest of the gene." is unclear, consider rephrasing.

We noticed that this statement, and broadly this whole paragraph, requires a detailed explanation that is more apt for the discussion. Hence, we have restricted it to a factual report, and merged the more interpretative parts to the paragraph in Discussion where we discuss LD.

In the sentence "This makes it conservative regarding SNPs with many interactions, like those mapped to hub genes in the PPIN.", what does "conservative" mean?

We replaced that expression by the more explicit “This raises the burden of proof required to select SNPs with many interactions, like those mapped to hub genes in the PPIN.”.

Define iCOGS array.

We have quoted the relevant part of the methods.

The end of the section 2.6 might belong to the discussion

In accordance to our answer above, we have moved this part as well to the Discussion.

Section 2.7
The sentence “However, although this new network exhibits similar global properties as the previous one, the lack of stability results in different genes being selected.” is unclear, consider rephrasing.

We have replaced the aforementioned sentence by “Despite these similarities, the consensus and the stable consensus solutions include different genes.”. We find this more appropriate, since we elaborate about the instability in the Discussion.

Discussion
In the sentence “Most of the network methods produced a relevant subset of biomarkers”, specify which methods.

We enumerated them.

In the sentence “Despite these similarities, the solutions were notably different.”, do you refer to the solutions found by all the methods?

Yes. We made it clearer: “Despite these similarities, the solutions of the methods were notably different.”.

In the sentence “While this deepens our understanding of the disease and provide biological hypotheses, they require further analyses”, give some examples of such analyses.

We added “For instance, we had to examine the centrality of the selected genes to discern the extent to which that property is driving the selection of each gene.”.

The sentence “Crucially, the consensus was as unstable as the tested methods, while the stable consensus shared these properties while accounting for instability.” is unclear, consider rephrasing.

We replaced that sentence by: “Importantly, while the consensus approach was as unstable as the individual network-guided methods, the stable consensus network retained the ability to provide compelling hypotheses and had better stability.”.

“Another source of instability might be the redundancy inherent to biological networks” => why ?

To explicit that, we replaced that fragment by the following: “Another source of instability might be the redundancy inherent to biological networks, since they are subject to an evolutionary pressure to avoid single points of failure. Hence, biological networks will often have multiple paths connecting two high-scoring nodes.”.

“In section 2.3, we highlight ambiguities …”, I’m not sure 2.3 is the correct section and the following sentences are unclear.

We do mean that section, specifically the case of HSPA1A, HSPA1B, and HSPA1L as we mention in the next sentence. It is a cluster of three genes that have described PPI between their proteins. Yet, they are so close in the genome, that 9 of the SNPs map to all of them.

We have clarified the remaining sentences in that paragraph.

The “presented case” => which one ?

We specified it in the text: “the case involving the nearby genes *HSPA1A*, *HSPA1B*, and *HSPA1L* is paradigmatic”.

“more resilient to them” => who ?

We split that long sentence into two. The relevant bit was rephrased as into “But the opposite case is also true: network methods might disregard such genes, despite their strong association with the phenotype, when they are functionally unrelated.”.

Comparison with the same approach on a larger network containing high-throughput and literature curated interactions. If the results are the same, how can they support the use of only interaction from HT experiments”? If the circular reasoning would be a problem, then the results would be different, no?

This observation aims to contribute to the ongoing discussion about which network is more apt. Indeed, we observe that, in our experiments, the results change little when using both LC and HT interactions. Yet, since we show that network methods have a strong preference for highly central nodes, we give the general recommendation of preferring HT networks. Simply as a cautionary step.

In the sentence “NEK10 (P-value 1.6 × 10 -5 , located near SLC4A7) or POU5F1B, were absent from the network”, are you talking about the Protein-Protein Interaction Network (PPIN) or the results you obtained?

It refers to the PPIN; we clarified that in the text.

If it is about the PPIN, why didn’t you use a different one?

We agree that selecting the PPIN that best describes a chosen set of genes would be a valid criteria for targeted studies. Yet, given the methodological nature of our work, we preferred the HINT PPIN. HINT compiles and curates 8 other databases. Thanks to that, at the time of its publication, it contained more PPIs than any of the 8 databases individually. Additionally, multiple PPI detection techniques are prone to false positives. HINT’s curation reduces the number of false positives by manually checking the interactions from high-throughput experiments, and including only replicated interactions in literature curated experiments. Lastly, it provides a convenient way of separating unbiased interactions (those coming from high-throughput studies) from biased interactions (those coming from low-scale studies, and targeting already well-known genes). This was important to make sure that the network we use is an unbiased sample of the actual human PPIN.

Specify phenotype in the sentence “However, on average protein-coding genes absent from the PPIN are less associated with this phenotype”.

Thanks, we did so.

In the sentence “As we are using interactions from high-throughput experiments, such difference…” it is unclear what the word “difference” refers to.

We have rewritten the sentence to make it clear it refers to the previous sentence: “This cannot be due to well-known genes having more known interactions because we are only using interactions from high-throughput experiments.”

The sentence “On the other hand, Huang et al. [27] found that the best predictor of the performance of a network for disease gene discovery is the size of the network, which supports using the largest amount of interactions.” is unclear, consider rephrasing.

We rewrote the sentence as follows: “On the other hand, Huang et al. found that larger networks were more useful than smaller networks to identify disease genes. This would support using the largest networks in our experiments.”.

Add the name of such algorithms to the sentence “... P-value precision limited to the number of permutations [43]), and other algorithms [43, 29, 58]...”.

We mentioned PEGASUS, SKAT and COMBAT in the new version of the manuscript.

Materials and methods

The method section is clearer than the result section. Pay attention that some details are not given in the method section but elsewhere in the manuscript (for instance the used p-value thresholds...), and that not all the method sections are quoted in the results when necessary.

Thanks, we agree with this observation. We addressed this by adding additional sections in the methods (*SNP- and gene-based GWAS; Comparing SNP-methods to gene-methods, and vice versa; Computational resources*), completing missing information, and making sure that all the relevant sections are quoted when needed.

Section 4.1

Syntax: “We focused on the 2 577 samples of European ancestry, out of which 1 279 are controls and 1 298 are cases.”.

Thanks, we fixed it.

Section 4.3

4.3.1

The sentence “For the former, we considered any gene that can be mapped to any of the selected SNPs as selected as well.” is unclear, consider rephrasing.

We added a new section “Comparing SNP-methods to gene-methods, and vice versa” which, hopefully, explains this more clearly.

4.3.3

“And they were designed to run efficiently on networks…” “they” is used to refer to 2 different things in the same sentence

Thanks, we fixed the error.

4.3.4.

The sentence “The authors propose a transformation of the genes’ P-value into a score that is negative under no association with the phenotype, and positive when there is.” seems to be incomplete.

Thanks, we replaced that sentence by “The authors propose a transformation of the genes’ P-value into a score that is negative under weak association with the phenotype, and positive under a strong one.”

Add the meaning of “equilibrium” to the sentence “This process continues until equilibrium is reached...”.

Thanks, we clarified that replacing that fragment by “This process continues until a stationary state is reached, in which the temperature of nodes does not change anymore,...”.

Section 4.6.
The code lacks comments and it is difficult to reproduce the results from the paper. Check broken links (I found this one https://github.com/hclimente/gwas-tools/blob/master)

Thanks for this suggestion. We tried to make the genewa repository more welcoming and easy to navigate. Additionally, we explain how the results of the paper were generated.

Supplementary materials
Fig. 2.
The x-axis labels from 18 to 23 are broken in the three subfigures.

We fixed the overlap by omitting the labels 19 and 21. Additionally, we fixed a similar issue on Figure S10.

Fig. 4.
OFD1 and COPS5 are not highlighted in (B)

We only color in pink known breast cancer susceptibility genes, both in panels A and B, but not *OFD1* and *COPS5*. We have clarified that in the legend.

Fig. 5.
The gene names (Y-axis) are hard to read, increasing space between names.

We noticed that that panel is quite low in information density, hard to read, and ultimately not needed, since the same information can be obtained from the supplementary files provided in NDEx. Hence, we removed it.

Fig. 7.
Define all abbreviations.

We expanded all the abbreviations in the axis text.

Are there more than 5K protein coding genes missing in your PPIN? Did you consider using another one?

We did consider using other PPIN. Yet, we decided against it for the reasons outlined in our previous answer.

Fig. 8.
Add legend in a separate box to improve readability.

Thanks, it did unclutter the image.

Reviewer #3: Network-based analysis of GWAS results is a potentially powerful approach for placing individual SNP/gene associations in the scaffold of molecular relationships to both improve interpretability and to the ability to identify marginally significant genes connected with significant genes. In this study, the authors perform an extensive analysis applying five methods for network-based GWAS analysis to study the associations from the GENESIS breast cancer study. The manuscript is written very well for the most part. The Introduction and Methods are particularly excellent as is the authors' effort to release their analysis code. This is a valuable study but there are several concerns that need to be addressed.

1. The authors bring up several times the point that network-based methods tradeoff statistical significance for biological relevance.
- First, the logistic regression (LR) models based on the genes/SNPs from the network-based methods are not that predictive. Second, the original LR model they build using all SNPs also has SNPs with high p-values, which is cited as a motivation for using network-based methods. So, neither the motivation nor the benefit of this "tradeoff" is apparent.

We find that network methods do "no worse" than LR in terms of classification accuracy, while providing an interpretable solution. Additionally, when such high P-value genes are added, there is a rationale for that: they are included because of their topological properties (high centrality, consistently affected neighborhood), as we point out at multiple points in the article. Hence, all things considered, a network-based approach is preferable. We have made this more explicit in the Discussion: "Hence, in this case network methods were preferable to the logistic regression classifier, since they do "no worse" while providing an interpretable solution."

- Similarly, why is solution subnetworks having lower p-values than the whole network (despite containing genes with higher p-values) exemplifying this tradeoff. The whole network contains the subnetwork and subnetworks of similar sizes can easily have lowe p-values by random chance.

That the solution networks have lower p-values than the entire network does not really illustrate the tradeoff between selecting statistically significant SNPs/genes and selected connected genes. Indeed, what matters for this tradeoff is that the solution networks have larger p-values than a classical GWAS: non-significant SNPs/genes are selected. We apologize if this was confusing.

2. In Table 1, please include the number of genes from each method that is included in the consensus network.

Thanks for this suggestion. We tried to convey this information through a column with the Pearson correlation with the consensus, in that same table. However, we find that this statistic might be more informative to the reader, so we replaced the former by the latter.

3. Association with network centrality:
- Genes in the four solution subnetworks exhibiting high between centrality: The dependence with betweenness shown in Table 1 seems to be a function of the number of genes in the subnetwork.

- Plus, in general, the association with high centrality genes is a chicken-and-egg problem.

- The same goes for the observations about the relationship with degree centrality (Figure 4B).

- The only way to check all these associations is to run all these analyses on a randomized PPI (where the edges have been shuffled while preserving node degrees) and compare to the real associations.

Thanks for this suggestion, it is indeed a relevant and straightforward analysis. We have added the section “Network rewirings” detailing the experimental setup, and included the results in section “Network topology and association scores matter, and might lead to ambiguous results”. Additionally, we added these considerations to the Discussion.

4. Figure 1:
- Figure 1C Precision-Recall analysis (comparison to BCAC): Is this analysis restricted to the set of common SNPs/genes?

All the comparisons between BCAC and GENESIS in the paper, are restricted to the set of common genes (those which have a VEGAS2 output in both datasets) and SNPs (those which passed QC in GENESIS). However, to compute the gene scores we used all 13,250,641 SNPs available on BCAC. We precised this in Section “Comparison to state-of-the-art”.

What is the interpretation?

Our main highlight is that recall is much higher for network methods than for a conventional χ^2 GWAS analysis, while having a much lower sample size. Since the χ^2 + Bonferroni procedure is conservative by design, precision gets worse. If BCAC was a perfect ground truth, the high recall - low precision of network methods would be preferable for exploratory analyses. Especially since network methods recover not only potentially causal genes, but also their functional context and a plausible mechanistic explanation. In fact, the omnigenic model considers that many causal genes are not discoverable through GWAS studies, although they all are interconnected in the network. In that regard, precision might be underestimated for network methods. Indeed, we observe in our results that genes like *COPS5* are also likely to play a role in breast cancer susceptibility, despite their high GWAS association P-value.

Both recall and precision here seem to be very low for all of the methods. The axes are a little confusing and also should be switched.

Thanks for noticing, we flipped Recall to the x-axis, and Precision to the y-axis, on both Figures S5 and S10.

Further, this is a chart talking about how BCAC genes relate to the methods. BCAC was never mentioned or defined in these early sections, so this does not make sense until one looks at this after reading much more of the paper. Same goes for BCAC mentioned in Figure 2.

Thanks, this is indeed a bad presentation of the methods on our side. We moved that panel to S5 Fig. With respect to Fig 2, we have quoted the BCAC part of the methods. With the same logic, we have added that reference to Fig 5.

- Comparing the network results to BCAC is a very useful validation metric. It would also be useful to run the network methods on the BCAC results themselves and compare the resulting networks from the different methods.

We considered that in the past, but decided otherwise because of three reasons. First, and foremost, we do not have access to the BCAC genotypes, only to summary statistics. This severely impairs our ability to conduct many of the most interesting experiments in the paper, which require subsampling. Second, this paper has a strong methodological component. In that regard, we found it interesting to explore a dataset having a “ground truth” to compare our findings. Lastly, the GENESIS dataset presents desirable properties: lack of differential population structure, well-characterized inclusion criteria, and focus on BRCA1/2 negative patients. And, notably, it is under-studied. In that regard, we found it important to study French population-specific biomarkers.

- Finally, it would be worth separating out figures relating to the BCAC comparison to the network methods from previous figures and putting them into this section. It would be more organized, make more sense rather than randomly seeing BCAC comparison in figure 1 where we are just being introduced to the network methods themselves.

Thanks for this suggestion. We have reorganized the panels of the figures to match better the text.

5. Figure 3:
- 3A: Are the lines standard error in each direction?

Yes, they are.

How can there be standard error in the direction of active set size since this seems to be fixed?

The active set is the of SNPs selected by the L1-penalized logistic regression, among the SNPs selected in one run of a network method. Its size is not set up by us, or directly determined by the initial number of SNPs, but set up by cross-validation by the algorithm itself. Notably, even different runs of L1-penalized logistic regression on the same data will lead to different cross-validation splits, and hence, might lead to different active sizes.

What is the point of the grey dotted line?

That line is meant as a visual indication of the upper limit of the active size (i.e. the total number of SNPs considered by the algorithm). We clarified that in the legend.

- 3B: Patient classification using selected SNPs. This analysis is not helpful. The authors acknowledge that they do not expect to separate cases from controls just using genetic data. And, as expected the sensitivity and specificity values are all pretty close to 0.5 and very close to each other. So, this analysis is not useful.

Although after the fact it would seem that, indeed, this is a pretty untelling analysis, we think it adds value to the paper. Particularly, we believe a significant number of readers might wonder what is the predictive capability of the selected genes / SNPs. Hence, we propose keeping it in the paper as a supplementary figure (Fig S5B).

- 3C: Does not have LEAN.

LEAN did not select any gene in any of the subsamples; in consequence, we excluded it from that panel (from the figure caption: “As LEAN did not select any gene, it was excluded from all panels except D.”).

Isn’t it intuitive that the more genes a network selects, the more stable that network is likely to be?

We believe that is a valid concern. Indeed, it is one of the criteria for stability measures discussed in Nogueira and Brown. They show how some measures are biased by it, but Pearson’s correlation is not one of those. In fact, we observe the opposite in our experiments, in which heinz and $\chi 2$ + Bonferroni are the most stable methods.

Saying LEAN is stable when it never gets any significant genes seems to be a stretch. It is consistent in that it never gives results.

In retrospect, we agree it is not the best description for LEAN. We have replaced that by “LEAN did not produce any solution in any of the subsamples.”

- 3D: Analysis of runtime is a little interesting but not useful at all. Runtime matters in the case of applying methods to a large number of traits/diseases. Since the application is to a single disease of interest, it should be reasonable if a method run for a day if it also returns reasonable/useful results.

We would argue that it is indeed useful. Since most practitioners will not be, at first, familiar with a particular method, they might need to iterate a few times until they get satisfactory results. That involves everything from fine-tuning parameters and getting the inputs in the right format to fixing a previously overlooked quality control step and re-running all the analyses. In that sense, if we imagine two methods which produce comparably useful results, a method like heinz which takes seconds to run is much more interesting than a method like HotNet2, which takes days.

Also, if the additional 119,980 sec for VEGAS2 is a constant, then why does adding it to the method runtime result in different distances between the corresponding triangles and circles in Figure 3D.

This is a consequence of using a logarithmic scale to represent time. This also allows us to visualize whether the time required to run a method is dominated by VEGAS2 (which you don't need to run again if you're using several methods).

6. Figure 4:
- Why is 4A labeled as a drawback?

We use figure 4A to show that the topology of dmGWAS' solution is not informative of gene importance in GWAS. If one naively examined that solution and looked at the modules, or the most central genes, they would prioritize a cluster of genes that are very weakly associated to breast cancer susceptibility. In our view, this low signal-to-noise ratio is a drawback, since it can lead to wrong conclusions.

- Doesn't it make sense in 4B that most genes would have a low centrality? Most genes are not central hubs in networks. Is it necessarily a bad thing that there are significant genes that do not have a high centrality degree?

Indeed, that's not a bad thing by itself. However, as we suspected, and our new rewiring experiments confirmed, network methods have a tendency to select highly central nodes. Provided that those genes are very low association scores to breast cancer susceptibility, one questions if including those genes in the solution is, itself good or bad. Since these hubs are used by network methods to connect far apart parts of their solutions, maybe a better solution is one in which these unrelated subnetworks stay separated. Or one that connects the two parts via a more relevant mechanism. Yet, as our new rewiring experiments show, network methods will tend to include these genes, even when the network topology is randomized.

- 4C seems very oddly scaled. One assumes that each of these is a chromosome, and it makes it look like the genomic regions from each chromosome are the same size. It also seems very unclear what the background color means here. Also for example in chromosome 5 here, how many genes are being looked at? It looks like there are at least 3 genes here, but that is not clear at all. This also does not seem to coincide with the results from the SConES network described in previous sections

That panel was meant to show both the overlap between the solutions of different versions of SConES, as well as whether they overlap with genes or not. We agree that in its previous form it was not helpful, maybe even confusing. Hence, we have replaced it by a figure that hopefully will do a better job at illustrating these two points.

7. Figure 5:
- In Figure 5B, add another boxplot for genes picked by just one method.

We moved this panel to Fig 1, since reviewer 1 was also interested. We added both the genes selected by one and three methods, and those not selected by any.

- Figure 5C is not cited in the text.

We agree with your comment below. We have removed Figure S5C.

8. Pathway enrichment analysis: Is the universe of genes should also be intersected with genes with at least one annotation in Reactome.

We have reviewed ReactomePA's *enrichPathway* code (here), which in turn calls DOSE's *enricher_internal* (here). Our understanding is that the latter indeed intersects the gene universe we provide and all the genes in Reactome on line 60.

9. Figure S5C is very misleading. There are only 4 familial BC genes. This is not a very good image in general and does not tell us anything.

We agree. We removed it.

Is there a comment on S5D where the familial genes don't really have much of a trend?

We find it difficult to comment on the familial breast cancer genes only, given that there are only 5 of them, it is not clear one can discuss whether or not they follow a trend. However, it is interesting to note that the known familial breast cancer genes recovered by the network approaches are included either because they have low p-values or because they have moderate (non-significant) p-values but higher centrality.
