# Supplementary material for: Boosting GWAS using biological networks: A study on susceptibility to familial breast cancer
Source: PLoS Comput Biol. 2021 Mar 18;17(3):e1008819. doi: 10.1371/journal.pcbi.1008819 (PMC8009366; doi:10.1371/journal.pcbi.1008819)
Supplement: Attachment — Submitted filename: GENESIS Answer to reviewers 2.pdf [file pcbi.1008819.s019.pdf]

Dear Dr. Roy, dear reviewers,

Thank you again for your patience, consideration, attention to detail, and contribution to enhancing this work. As before, we have addressed your comments below (responses in blue). We attach the article to this letter as well as a marked version with all the changes.

We summarize here the main changes:

- Title and abstract rewritten
- Included SNP and gene summary statistics as supplementary files (Previously, these files were available only on the GitHub repository.)
- Used normalized betweenness as a measure of centrality, replacing both (unnormalized) betweenness and degree centrality
- Added a reference to Climente-González et al. (2021), where we introduce martini, the implementation of SConES that we use
- Added a line on Lonjou et al. (2021), a recent article on GENESIS which displays slightly better classification accuracy than the presented
- Fixed several issues with figures regarding missing text and overlapping labels
- Fixed typos and wording

Reviewer #2: The authors provide detailed responses to our previous comments as well as a revised version of their manuscript highly improved. The results are now much more clear and easy to follow, the interest of the work more straightforward, and figures have been improved.

Minor comments

Abstract and title are the two remaining points where there is still room for improvement in clarity. The title is a bit long, and the abstract has some of the previous weaknesses. In particular "project-agnostic" is unclear.

We agree with the reviewer's opinion. We have completely rewritten the abstract and changed the title.

There seems to be problems in the figure numbering, as in the figure pages, the figure number title does not match with the figure number in the link, starting at figure 2, which is linked as figure3.

We apologize for this; we kept the original figure naming, despite the changes in the text. We renumbered the figure names accordingly.

Introduction

"and aggregate them ..." is this really an aggregation? Aggregation could mean the union of the solution, whereas the consensus is using the intersection of the solutions.

That looks more accurate; we replaced it by “studied their intersection.”

Methods

The methods section describes 2 network topology measures, but only one median (betweenness) is presented in Table2 and discussed in the corresponding section of the results. Similarly, Figure 4 presents only the centrality measure but not the betweenness.

Thank you for this relevant observation. Degree centrality is a local measure of centrality, which informs about the local connectivity. Betweenness is a global measure that informs about the gene’s role on the whole network. To a certain extent, they give similar information, but the first one has a straightforward interpretation, which is why we favored it in the figures. However, we believe betweenness is more relevant for this work. For this reason, and for consistency, we will use only betweenness as a centrality measure. To make it easier to interpret, we will use its normalized version.

This led to the following changes: we describe only (normalized) betweenness in the methods, and we only report that measure across the article.

Network rewiring
“We only applied only four methods”, the word “only” is repeated.

Thanks, we fixed it.

2.4
"gene solutions" is unclear

We rephrased that sentence as “We searched for pathways enriched in the solution of each network method.”.

3.2
A remaining unclear point is if the gene functional enrichment analysis is done individually on each subnetwork solution, or on the union of the genes from the different solutions.

To make this clearer, we specify in 2.4 that we perform a pathway enrichment analysis for each of the solutions: “We searched for pathways enriched in the solution of *each* network method.” Additionally, we changed the first sentence in 3.2 to “Lastly, pathway enrichment analyses on each method's solution (Section 2.4) also revealed similarities and differences between them.’

"submodule" is unclear, is it a subnetwork solution?

It involves a group of interconnected proteins. Given that we discuss a solution, in which proteins are already interconnected, we removed this term.

3.6
Last part of the section, "While the solutions on those ... (Fig4C)", isn't it Figure 4D?

That is correct; we fixed it.

Some parts of the section “Network topology and association scores matter and might lead to ambiguous results” seem more appropriate for the discussion.

We agree with that observation. However, we think that those parts are essential to articulate this section and give the reader the broader context needed to understand these results.

Figures

Figure 1
C. The networks corresponding to HotNet2 and SconES GI are overlapping with the legends.
D. Y-axis labels are missing

Thanks, we fixed both issues.

Figure 2
A. Y-axis labels are missing and Fig2A it is not referenced in the text

We added the missing x- and y-axis labels. We added references to Figures 2A and 5A, which were never referenced either.

Figure 2B and 2C could be merged, the same information can be provided in the same network representation. Same observation for Figure 5B and 5C.

We agree that they are, to a certain extent, redundant. However, after trying several approaches that looked overwhelming, we realized that these information-dense figures are more useful to the reader if kept separate.

Figure 3
D. The axis’ legends (true positive and false positive rates) and the figure legend (true positive and true negative rates) do not match.

Indeed, we fixed it: the legend now reads “false positive rate” as well.

Figure 4
B. The legend of USHBP1 overlaps another one.

Thanks, we fixed it.

D. Not referenced in the text

We had mistakenly referenced panel C instead of D (addressed above).

Figure 5
A. Y-axis labels are missing

Thanks, we added them.

C. There is a line between the pink DNAJC1 legend and its corresponding node, which is a bit misleading since it is the only occurrence.

We made sure no such lines appear in Figure 2 or Figure 5.

Figure S2.
C. Why none of the dots above the red line are labeled?

Panel A represents a conservative procedure, where we can label all the SNPs above the significance threshold without overwhelming the reader. When we labeled every selected SNP in panel C, however, the result was unreadable. On the other hand, labeling only the SNPs above the line might give the wrong impression that the Bonferroni threshold plays a role in Lasso's selection. Hence, we prefer to leave it as is.

Figure S6. LEAN seems to be labeled as SconES GI or vice versa,

Indeed, we showed incomplete SConES GI's results (the 6/100 runs that got completed in time for the last submission). LEAN did not produce any results i.e., no subnetwork was significant in any rewiring. We have specified that in the methods and corrected the figure's caption.

plus it is referenced before figure S5.

Indeed, we switched S5 Fig and S6 Fig.

Figure S7.
B. Seems to be truncated on the right

Indeed, we fixed it.

Figure S10. LC is not defined,

LC refers to a "Literature curated (interaction)," a term we borrowed from the HINT article. We do not define LC, but we do define the only abbreviation that contains it ("HT + LC"): "on

high-throughput (HT) interactions to the benchmark on both *high-throughput and literature curated interactions* (**HT+LC**).”.

plus it is referenced before figure S9.

Indeed. We switched S9 and S10.

Figure S11. Missing (the download link shows figure S10)

We apologize for that oversight; we accidentally overwrote the S11 file. We made sure we uploaded the correct one this time.

Discussion
“To overcome these limitations while exploiting the each method’s strengths…” syntax error, remove “the”

Thanks, fixed.

Acknowledgments
"clinics" duplicated

Thanks, fixed.

The text is not justified (alignment)

That seems to be PLoS’ preferred text alignment.

Reviewer #3: The authors have satisfactorily addressed all the questions and concerns raised by the reviewer.
